# Phenotypic and neuropathological heterogeneity in autosomal dominant familial Alzheimer's disease, and implications for amyloid‐immunotherapy

**DOI:** 10.1002/alz70857_099386

**Published:** 2025-12-24

**Authors:** Natalie S Ryan, Clíona Farrell, Moneeb Nasir, Nanet Willumsen, Ricardo G Pires, Charlotte Burdon, Frances K Wiseman, Tammaryn Lashley, Lei Liu, Jasmeer P. Chhatwal, Nick C Fox

**Affiliations:** ^1^ UK Dementia Research Institute at UCL, London, United Kingdom; ^2^ Dementia Research Centre, UCL Queen Square Institute of Neurology, London, United Kingdom; ^3^ Queen Square Institute of Neurology, University College London, London, United Kingdom; ^4^ Functional Unit of Neuroradiology, Department of Medical Imaging, Unidade Local de Saúde de Coimbra, Portugal, Coimbra, Portugal; ^5^ Ann Romney Center for Neurologic Diseases, Department of Neurology, Brigham and Women's Hospital, Harvard Medical School, Boston, MA, USA; ^6^ Brigham and Women's Hospital, Harvard Medical School, Boston, MA, USA; ^7^ Massachusetts General Hospital, Harvard Medical School, Boston, MA, USA

## Abstract

**Background:**

In autosomal dominant Alzheimer's disease (ADAD), mutations in *APP*, *PSEN1* and *PSEN2* alter amyloid‐β processing, driving production of longer, aggregation‐prone amyloid‐β petides that deposit in parenchyma as plaques and blood vessel walls as cerebral amyloid angiopathy (CAA). However, different mutations vary in the amyloid‐β isoforms produced, the type and severity of amyloid‐β plaques and CAA, and clinical manifestations. As CAA is a risk factor for amyloid‐related imaging abnormalities (ARIA) during anti‐amyloid‐β immunotherapy, investigating relationships between clinical, genetic and neuropathological heterogeneity may have relevance for understanding the natural history of the disease and responses to therapy.

**Methods:**

50 individuals with ADAD underwent post‐mortem brain donation to the Institute of Psychiatry or Queen Square Brain Bank (QSBB); 37 with *PSEN1* mutations, 13 with *APP*. Clinical phenotype data were reviewed and frontal cortex sections stained immunohistochemically with a pan amyloid‐β antibody. Blood vessel counts were used to determine the frequency and severity of CAA in leptomeninges and parenchyma. The 29 QSBB cases were also stained using a lecanemab‐biosimilar antibody (lecanemab‐BS), and immunofluorescence co‐staining was carried out for lecanemab‐BS, amyloid‐β_40_, amyloid‐β_42_ and amyloid‐β_43_. 10 of these individuals had undergone MRI including susceptibility‐weighted imaging (SWI) sequences during life. Relationships between clinical, imaging and neuropathological features were explored.

**Results:**

Non‐amnestic cognitive presentations and atypical clinical features (pyramidal, extrapyramidal and cerebellar signs) were more common with *PSEN1* than *APP* mutations. All cases demonstrated end‐stage AD pathology, however the frequency and severity of CAA varied considerably. Lecamemab‐BS labelled amyloid‐β plaques (diffuse and dense‐core) in all cases, and extensively labelled amyloid‐β in blood vessels when CAA was present. More severe CAA was associated with greater lecanemab‐BS binding. Four of the 10 individuals with SWI had one to three microbleeds on MRI. These four all had moderate‐severe CAA post‐mortem, however, so did five of those without microbleeds.

**Conclusion:**

Lecanemab‐BS bound to amyloid‐β plaques and CAA in cases of ADAD with a variety of mutations and clinical features. The increased lecanemab binding in blood vessels with more severe CAA highlights the need for more sensitive biomarkers of CAA during life and provides further incentive for treating early in the disease.

